# Artificial intelligence utilising corneal confocal microscopy for the diagnosis of peripheral neuropathy in diabetes mellitus and prediabetes

**DOI:** 10.1007/s00125-021-05617-x

**Published:** 2021-11-21

**Authors:** Frank G. Preston, Yanda Meng, Jamie Burgess, Maryam Ferdousi, Shazli Azmi, Ioannis N. Petropoulos, Stephen Kaye, Rayaz A. Malik, Yalin Zheng, Uazman Alam

**Affiliations:** 1 Department of Eye and Vision Science, Institute of Life Course and Medical Sciences, University of Liverpool, Liverpool, UK; 2 Institute of Life Course and Medical Sciences and the Pain Research Institute, University of Liverpool and Liverpool University Hospital NHS Foundation Trust, Liverpool, UK; 3 Institute of Cardiovascular Science, University of Manchester and Manchester Diabetes Centre, Manchester Foundation Trust, Manchester, UK; 4 Weill Cornell Medicine – Qatar, Doha, Qatar; 5 St Paul’s Eye Unit, Royal Liverpool University Hospital, Liverpool, UK; 6 Division of Endocrinology, Diabetes and Gastroenterology, University of Manchester, Manchester, UK

**Keywords:** Artificial intelligence, Convolutional neural network, Corneal confocal microscopy, Deep learning algorithm, Diabetic neuropathy, Image segmentation, Ophthalmic imaging, Small nerve fibres

## Abstract

**Aims/hypothesis:**

We aimed to develop an artificial intelligence (AI)-based deep learning algorithm (DLA) applying attribution methods without image segmentation to corneal confocal microscopy images and to accurately classify peripheral neuropathy (or lack of).

**Methods:**

The AI-based DLA utilised convolutional neural networks with data augmentation to increase the algorithm’s generalisability. The algorithm was trained using a high-end graphics processor for 300 epochs on 329 corneal nerve images and tested on 40 images (1 image/participant). Participants consisted of healthy volunteer (HV) participants (*n* = 90) and participants with type 1 diabetes (*n* = 88), type 2 diabetes (*n* = 141) and prediabetes (*n* = 50) (defined as impaired fasting glucose, impaired glucose tolerance or a combination of both), and were classified into HV, those without neuropathy (PN−) (*n* = 149) and those with neuropathy (PN+) (*n* = 130). For the AI-based DLA, a modified residual neural network called ResNet-50 was developed and used to extract features from images and perform classification. The algorithm was tested on 40 participants (15 HV, 13 PN−, 12 PN+). Attribution methods gradient-weighted class activation mapping (Grad-CAM), Guided Grad-CAM and occlusion sensitivity displayed the areas within the image that had the greatest impact on the decision of the algorithm.

**Results:**

The results were as follows: HV: recall of 1.0 (95% CI 1.0, 1.0), precision of 0.83 (95% CI 0.65, 1.0), *F*_1_-score of 0.91 (95% CI 0.79, 1.0); PN−: recall of 0.85 (95% CI 0.62, 1.0), precision of 0.92 (95% CI 0.73, 1.0), *F*_1_-score of 0.88 (95% CI 0.71, 1.0); PN+: recall of 0.83 (95% CI 0.58, 1.0), precision of 1.0 (95% CI 1.0, 1.0), *F*_1_-score of 0.91 (95% CI 0.74, 1.0). The features displayed by the attribution methods demonstrated more corneal nerves in HV, a reduction in corneal nerves for PN− and an absence of corneal nerves for PN+ images.

**Conclusions/interpretation:**

We demonstrate promising results in the rapid classification of peripheral neuropathy using a single corneal image. A large-scale multicentre validation study is required to assess the utility of AI-based DLA in screening and diagnostic programmes for diabetic neuropathy.

**Graphical abstract:**

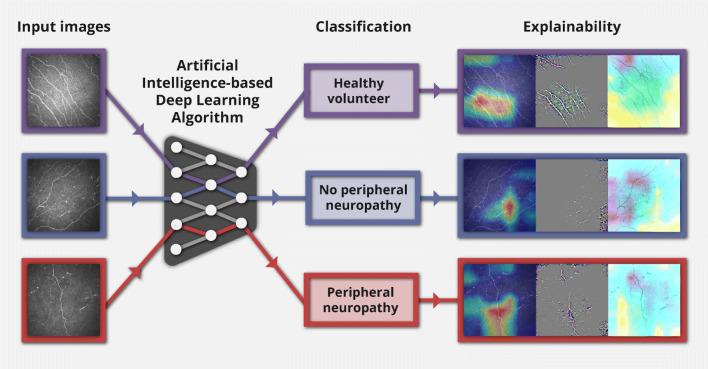

**Supplementary Information:**

The online version of this article (10.1007/s00125-021-05617-x) contains peer-reviewed but unedited supplementary material.

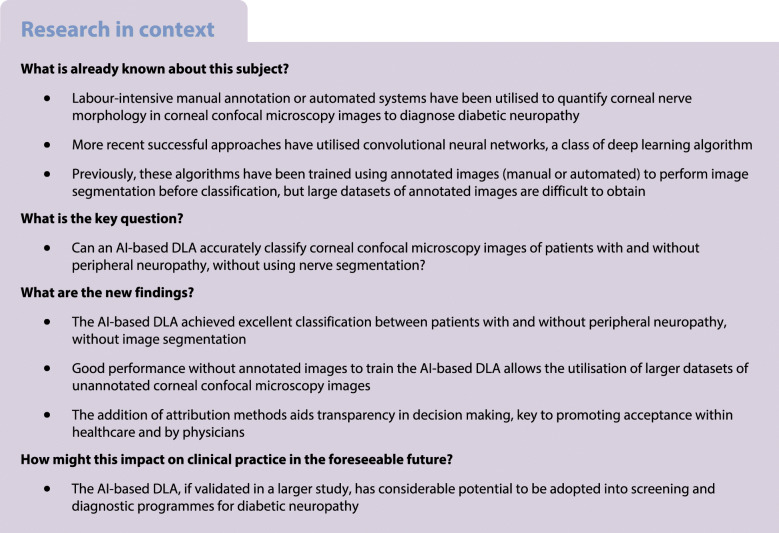



## Introduction

Diabetes mellitus had an estimated worldwide prevalence in 2017 of 451 million which is expected to rise to 693 million people by 2045 [1]. Neuropathy affects ~50% of people with diabetes and diabetes is the leading cause of neuropathy worldwide [2]. It results in neuropathic pain which impacts on quality of life and may lead to foot ulceration and amputation, with an excess premature mortality rate. Peripheral neuropathy has also been demonstrated in approximately 10% of individuals with prediabetes [3]. Given that prediabetes is projected to affect up to 587 million people (8.3% of the global adult population) by 2040, this represents a major burden on healthcare. Early diagnosis of diabetic neuropathy is essential to prevent progression [4] and subsequent morbidity and mortality rate [2]. A robust screening programme that incorporates reliable state-of-the-art technologies and biomarkers is required to deploy targeted screening for neuropathy in prediabetes and diabetes.

Current screening methods for diabetic neuropathy rely on neurological examination or 10 g monofilament which detect moderate to severe neuropathy affecting the large nerve fibres, yet small nerve fibres are the earliest to be damaged. Skin biopsy with quantification of intra-epidermal nerve fibres is the current reference standard to detect small fibre damage [4], but this method is invasive [5] and there are limited specialist clinical laboratories undertaking this procedure, making it unsuitable for population-level screening of peripheral neuropathy. In vivo corneal confocal microscopy (CCM) is a non-invasive, rapid and reiterative ophthalmic imaging technique that can quantify small nerve fibres in the cornea [4, 5]. Indeed, corneal nerve loss occurs in subclinical diabetic neuropathy [6], increases with the severity of diabetic neuropathy [7] and predicts incident diabetic neuropathy [8]. A large body of published data has shown that CCM can be used to diagnose and monitor progression of diabetic neuropathy [4, 9, 10]. Additionally, CCM detects nerve fibre regeneration in clinical trials of patients with diabetic neuropathy [5, 10], which precedes improvements in symptoms and neurophysiology [11]. Normative ranges have been established [12] and the corneal subbasal nerve plexus remains stable in healthy individuals over 3 years [13].

However, quantitative analysis of the subbasal nerve plexus requires reliable extraction of image features [14], and although manual segmentation of corneal nerve fibres is sensitive [15] and reproducible [16], it is operator-dependent and laborious. Dabbah et al. [14] developed an automated image analysis system using a dual model feature descriptor combined with an artificial neural network which correlated highly with manual measurements [17]. Chen et al. [18] further refined the automated software using either a neural network or random forest for classification and achieved a performance equivalent to that of manual annotation, combined with greater reproducibility and speed. More recently, advanced convolutional neural networks (CNNs), a class of deep learning algorithm (DLA), have been developed to enhance feature detection [19] and quantification of corneal nerve fibre morphology and have produced promising results [20–23]. Williams et al. [22] compared an artificial intelligence (AI)-based DLA with ACCMetrics [18] and demonstrated more consistent quantification of corneal nerve morphology with a superior diagnostic performance [22]. In a small dataset, Scarpa et al. [21] utilised a CNN on corneal nerve images (without segmentation) and classified individuals who were healthy or had diabetic neuropathy with an accuracy of 96%.

Despite providing accurate decisions comparable to human experts, the deployment of AI into medical practice has been partly hindered by its ‘black-box’ nature and the inability to provide the logic for the decision to end users. Thus, identifying the features by which the AI-based DLA classifies disease, in addition to the quantitative algorithmic performance, is key to promoting acceptance within healthcare and by physicians [24]. The primary modality used to explicate AI-based DLA within medical imaging diagnostics is attribution based, where the contribution to the output decision of each input feature is determined, allowing the generation of heat-maps known as attribution maps [24]. Gradient-weighted class activation mapping (Grad-CAM), Guided Grad-CAM and occlusion sensitivity are extensively used attribution methods which generate visual outcomes via attribution maps [25, 26].

The aim of this study was to develop and refine an AI-based DLA utilising image classification to identify healthy volunteer (HV) participants and individuals with prediabetes and diabetes with and without neuropathy, without using image segmentation. Grad-CAM, Guided Grad-CAM and occlusion sensitivity attribution methods were implemented to provide transparency and explanation of the AI-based DLA decision-making process.

## Methods

### Participants

All participants provided informed valid consent prior to assessments and the study was conducted in accordance with the Declaration of Helsinki. Ethical and institutional approvals were obtained before the participants completed the scientific protocol including CCM imaging. Other causes of peripheral neuropathy (except for diabetes/prediabetes) were excluded based on a comprehensive medical and family history and blood tests (immunoglobulins, anti-nuclear antibody, vitamin B_12_ levels, thyroid function tests). Prediabetes was defined using standard international criteria (WHO/ADA) (impaired fasting glucose, impaired glucose tolerance or a combination of both). Peripheral neuropathy was defined according to the Toronto Consensus on diabetic neuropathy, which defined confirmed diabetic neuropathy as a combination of an abnormality of nerve conduction studies and a symptom(s) and/or sign(s) of neuropathy [27]. Participant data upon which the peripheral neuropathy diagnosis was originally confirmed were available in 360/369 participants. These data were independently assessed by two authors (UA and MF) to determine the diagnosis. For any disagreement between authors, a third author (INP) made the final decision. The Cohen’s κ score, which measures inter-rater reliability, between UA and MF was 0.962, demonstrating almost perfect agreement.

### Image dataset and dataset preparation

**Fig. 1:**
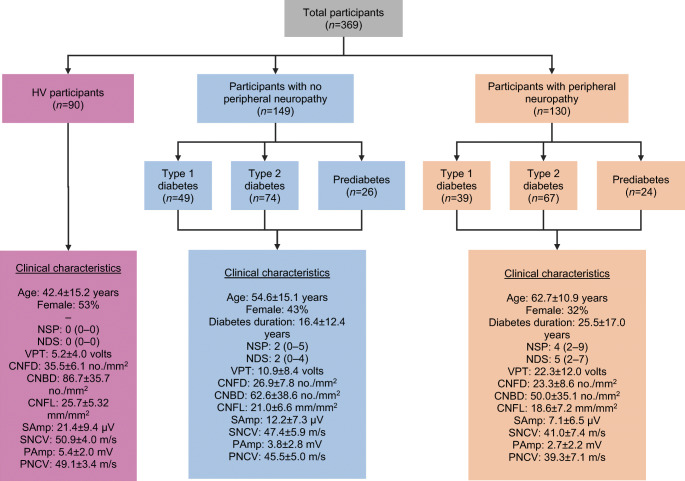
Flowchart of participant groups and clinical characteristics within HV participants, participants with no peripheral neuropathy and participants with peripheral neuropathy. Data are mean ± SD for age, diabetes duration, CNFD, CNBD, CNFL, VPT, SAmp, SNCV, PAmp and PNCV. Data are median (interquartile range) for NSP and NDS. People with confirmed peripheral neuropathy had greater neuropathic deficits with more signs (NDS) and symptoms (NSP), higher VPT and lower CNFD, CNFL, CNBD, SNCV, PNCV, SAmp and PAmp. People with peripheral neuropathy were older and those with diabetes had a longer duration of disease. CNBD, corneal nerve branch density; CNFD, corneal nerve fibre density; NDS, neuropathy disability score (score out of 10); NSP, neuropathy symptom profile (score out of 38); SAmp, sural nerve amplitude; SNCV, sural nerve conduction velocity; PAmp, peroneal nerve amplitude; PNCV, peroneal nerve conduction velocity; VPT, vibration perception threshold (score out of 50)

The dataset (Early Neuropathy Assessment [ENA] group, University of Manchester, UK) consisted of images of the corneal subbasal nerve plexus from HV participants and participants with prediabetes and diabetes (*n* = 369). The CCM images were captured, using a standard, internationally accepted protocol developed by the ENA group, at 400 × 400 μm (384 × 384 pixels) using a Heidelberg Retina Tomograph III using the Rostock Corneal Module (RCM; HRTII32-RCM) confocal laser microscope (Heidelberg Engineering, Heidelberg, Germany). To enable compatibility with the image analysis software, the images were exported in the BMP file format. The images used were from: HV (*n* = 90); type 1 diabetes with neuropathy (*n* = 39); type 1 diabetes without neuropathy (*n* = 49); type 2 diabetes with neuropathy (*n* = 67); type 2 diabetes without neuropathy (*n* = 74); prediabetes with neuropathy (*n* = 24); prediabetes without neuropathy (*n* = 26). There were 90 HV participants, 149 participants with no peripheral neuropathy (PN−) and 130 participants with peripheral neuropathy (PN+) (Fig. 1). Neuropathy data for each of the three groups are detailed in Fig. 1. In keeping with the neuropathic phenotype, people with confirmed peripheral neuropathy had greater neuropathic deficits with more signs (higher neuropathy disability score) and symptoms (higher neuropathy symptom profile), higher vibration perception threshold, and lower peroneal and sural nerve conduction velocities and amplitudes, corneal nerve fibre length (CNFL), corneal nerve branch density and corneal nerve fibre density. As expected, people with peripheral neuropathy were older and, in those with diabetes, there was a longer duration of disease.

Each of the CCM images was labelled with its respective class, ‘control’, ‘no neuropathy’ or ‘neuropathy’, allowing supervised training to occur. Out of a total of 369 CCM images, 245 (66%) were used in the training set, 84 (23%) in the validation set and 40 (11%) in the test set. Electronic supplementary material (ESM) Table 1 demonstrates the breakdown of participant groups within the training, validation and test sets. The distribution between groups was allocated randomly, using the Python package ‘random’ to generate a random number for each image. A random number was generated between 0 and 1; if it was between 0 and 0.1, the image was put into the test dataset; if it was between 0.1 and 0.3, it was put into the validation dataset; and if it was between 0.3 and 1, it was put into the training dataset. Each participant had up to seven CCM images; however, when all the images were used the AI-based DLA suffered significantly from overfitting. Therefore, a single image for each participant was selected at random. Data augmentation strategies were employed in the training of the algorithm, having been previously shown to increase the generalisability of AI-based DLAs [28], where additional training images were generated via images being either rotated between 0 and 90 degrees or flipped on their horizontal axis.

### Network architecture

ResNet is a residual neural network proposed by He et al. [29] at the 2015 ImageNet competition, where it achieved first place in the classification task. A ResNet network was developed as it overcomes the ‘vanishing gradient problem’ [30] by introducing skip (or shortcut) connections, where input from a previous layer can be transferred to the next layer without modification, allowing ResNet to have up to 152 layers [29]. Our ResNet-50 model comprises 50 layers, which culminate in a dense layer of 1000 neurons that has an applied softmax activation function. Two types of shortcut modules allow the ResNet-50 model to employ skip connections: convolution blocks and identity blocks. Convolution blocks contain a convolutional layer within the skip connection which results in the input dimensions being smaller compared with the output dimensions. Identity blocks do not contain a convolutional layer within the skip connection, meaning input and output dimensions are the same. In both shortcut modules, a 1 × 1 convolutional layer begins and ends the module, employing a bottleneck design to enable the reduction of parameters without degrading network performance.

**Fig. 2:**
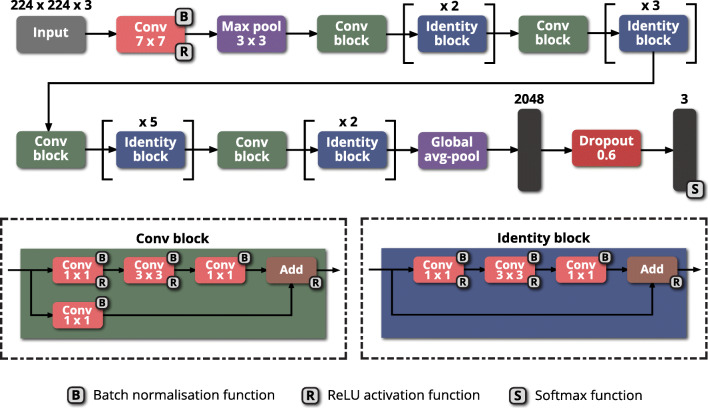
Diagram of the modified ResNet-50 architecture. Each pink rectangle corresponds to a convolutional layer, with the filter size given within. Each purple rectangle corresponds to a pooling layer, either maximum pool or global average pool. Each green rectangle corresponds to a convolution block. Each blue rectangle corresponds to an identity block. Each black rectangle corresponds to a dense layer. Each red rectangle corresponds to a dropout layer (dropout = 0.6). Avg, average; Conv, convolutional; Max, maximum; ReLU, rectified linear unit

A modified version of the ResNet-50 architecture (Fig. 2) was used to extract features from the images and perform classification. Modifications involved replacing the dense layer of 1000 neurons with one of 2048 neurons, adding a dropout layer with a rate of 0.6 after this layer and ending with a final dense layer of three neurons with the softmax activation function being applied to it, since there were three classes. The largest probability of three classes’ predictions (e.g., argmax) was used to determine the class label. The dropout layer was added to reduce overfitting, achieving this by randomly dropping layers and their connections during training, preventing layers from co-adapting, where one corrects the mistakes of other layers, but does not generalise to new data [31]. The initial weights of the model were pre-trained on the ‘ImageNet’ dataset [32], with weights in all the layers being set to be trainable.

### Additional models

Further experiments were conducted to allow comparison of the modified ResNet-50 model. We adopted the backbone of MobileNet and MobileNetV2 [33] to perform the comparison experiments under the same experimental setting. Note that the same modification was done with respect to the model structure. Compared with ResNet-50, MobileNet and MobileNetV2 are lightweight models that contain relatively fewer model parameters. This choice was made to demonstrate the effectiveness of model size in this work.

### Implementation details

Before training the model, we undertook pre-processing on input images. For example, we resized the image from 384 × 384 to 224 × 224 with the bilinear interpolation method. We increased the image channel from 1 to 3 through replicating along the channels. Additionally, we first scaled the image pixel values into [0–1] and then normalised the values in the range of [−1, 1] by using a mean value of 0.5 and an SD of 0.5 for three channels. The underlying motivations are threefold: First, due to the limited GPU graphic memory, we resized the input image into a lower size for training. Second, given the limited dataset size in this work, overfitting may be a potential issue during the model training. To address this issue, we increased the image channel size for fitting a pre-trained model on ImageNet [32]. Third, normalisation of pixel values can stabilise the training process and benefit optimisation [34].

The model was trained for 300 epochs (passes of the entire dataset) on the training datasets and evaluated on the validation datasets. The model was trained (245 images) and then used to predict the class of images in the validation dataset (84 images) to determine the validation accuracy. After each epoch, the model’s weights were altered via backpropagation and gradient descent, with the weights of the model achieving the highest validation accuracy being saved and applied to the test set (40 images—equal to 40 participants) to perform classification. Experiments were conducted with a batch size of 12, 24 and 36; learning rate of 0.01, 0.001 and 0.0001; and dropout rate of 0.6, 0.4 and 0.2. Hyperparameters were empirically set with a batch size of 12, learning rate of 0.001 and dropout rate of 0.6. The optimiser was stochastic gradient decent (SGD), and the loss function was cross entropy. Early stopping was set to monitor validation accuracy, which discontinued training if an improvement in validation accuracy did not occur after 100 epochs.

The model was developed, tested and trained within Python 3.7 (https://www.python.org/), Tensorflow 2.2.0 (https://www.tensorflow.org/; Google, Mountain View, CA, USA) and Keras 1.0.8 (https://keras.io/) on a high-end graphics processor, NVIDIA GeForce GTX 960M (NVIDIA, Santa Clara, CA, USA).

### Performance evaluation

A confusion matrix was developed to ascertain the AI-based DLA performance, displaying the true image classifications against the classifications predicted by the AI-based DLA. Using the confusion matrix, a classification report was produced displaying the widely used performance metrics precision, recall (also known as sensitivity) and *F*_1_-score. Precision is the proportion of true positive cases out of all the predicted positives which measures the effects of false-positives. Recall is the ratio of the predicted positives and total actual positives. *F*1 = 2 (precision × recall)/(precision + recall) and measures the trade-off between precision and recall. 95% CIs were generated to show statistical significance. In detail, 2000 samples of Clopper–Pearson interval [35] were used for precision, recall and *F*_1_. Fivefold cross-validation was done across all experiments to provide more robust results; the performance is reported as the mean of fivefold results.

### Attribution maps

The attribution method Grad-CAM utilises the gradients entering the final convolutional layer to generate a coarse attribution map, which demonstrates the areas in the image that have impacted the decision most [25]. Grad-CAM can be further combined with the fine-grained image to generate a high-resolution class-discriminative visualisation known as Guided Grad-CAM [25]. Occlusion sensitivity systematically occludes different areas of the input image with a grey patch, and monitors the effect of this on the classification [26]. A grey patch of 48 pixels was used in this study. Grad-CAM, Guided Grad-CAM and occlusion sensitivity were employed to generate attribution maps for each of the test images.

## Results

### ResNet-50 classification performance

The confusion matrix generated after the trained AI-based DLA had classified the test dataset (*n* = 40) is displayed in Table 1. All HV images (*n* = 15) were correctly detected by the AI-based DLA. Out of the PN− images (*n* = 13), 11 were correctly detected by the AI-based DLA, and two misclassified as HV images. Of the PN+ images (*n* = 12), ten were correctly detected, with one misclassified as PN− and one as HV.

**Table 1:** Confusion matrix report from modified ResNet-50 in HV, PN− and PN+

True class	Predicted class		
	HV	PN−	PN+
HV	15	0	0
PN−	2	11	0
PN+	1	1	10

Using the data demonstrated in the confusion matrix, a classification report (Table 2) was produced with the performance metrics described previously. In detecting HV images, the AI-based DLA had a recall of 1.0 (95% CI 1.0, 1.0), precision of 0.83 (95% CI 0.65, 1.0) and *F*_1_-score of 0.91 (95% CI 0.79, 1.0); for PN− images, the AI-based DLA had a recall of 0.85 (95% CI 0.62, 1.0), precision of 0.92 (95% CI 0.73, 1.0) and *F*_1_-score of 0.88 (95% CI 0.71, 1.0); and for PN+ images, the AI-based DLA had a recall of 0.83 (95% CI 0.58, 1.0), precision of 1.0 (95% CI 1.0, 1.0) and *F*_1_-score of 0.91 (95% CI 0.74, 1.0).

**Table 2:** Classification report from modified ResNet-50 in HV, PN− and PN+

Class	Recall (Sensitivity)	Precision	*F*_1_-score
HV	1.0 (1.0, 1.0)	0.83 (0.65, 1.0)	0.91 (0.79, 1.0)
PN−	0.85 (0.62, 1.0)	0.92 (0.73, 1.0)	0.88 (0.71, 1.0)
PN+	0.83 (0.58, 1.0)	1.0 (1.0, 1.0)	0.91 (0.74, 1.0)

Note: 95% CIs are given in brackets

### MobileNet and MobileNetV2 classification performance

Confusion matrices were also generated for MobileNet (ESM Table 2) and MobileNetV2 (ESM Table 3). Classification reports were produced based on these confusion matrices for both MobileNet (ESM Table 4) and MobileNetV2 (ESM Table 5). In detecting HV images, MobileNet had a recall of 1.0 (95% CI 1.0, 1.0), precision of 0.68 (95% CI 0.50, 0.87) and *F*_1_-score of 0.81 (95% CI 0.67, 0.93); for PN− images, MobileNet had a recall of 0.54 (95% CI 0.25, 0.82), precision of 0.88 (95% CI 0.57, 1.0) and *F*_1_-score of 0.67 (95% CI 0.36, 0.87); and for PN+ images, MobileNet had a recall of 0.75 (95% CI 0.46, 1.0), precision of 0.90 (95% CI 0.67, 1.0) and *F*_1_-score of 0.82 (95% CI 0.58, 0.96). In detecting HV images, MobileNetV2 had a recall of 0.87 (95% CI 0.67, 1.0), precision of 0.72 (95% CI 0.50, 0.93) and *F*_1_-score of 0.79 (95% CI 0.60, 0.92); for PN− images, MobileNetV2 had a recall of 0.62 (95% CI 0.33, 0.90), precision of 0.67 (95% CI 0.36, 0.92) and *F*_1_-score of 0.64 (95% CI 0.38, 0.84); and for PN+ images, MobileNetV2 had a recall of 0.75 (95% CI 0.46, 1.0), precision of 0.90 (95% CI 0.67, 1.0) and *F*_1_-score of 0.82 (95% CI 0.57, 0.97).

The ResNet-50 model had the lowest number of misclassifications (*n* = 4), followed by MobileNet (*n* = 9) and MobileNetV2 (*n* = 10). The ResNet-50 model also performed better than MobileNet and MobileNetV2 in all performance metrics across all classes. For instance, in detecting PN+, ResNet-50 achieved 10.7%, 11.1% and 11.0% higher recall, precision and *F*_1_-score than both MobileNet and MobileNetV2.

### ResNet-50 attribution maps

**Fig. 3:**
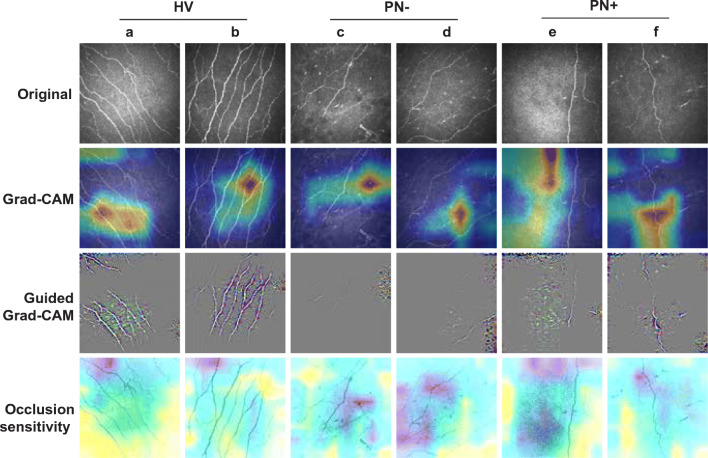
Attribution map results from ResNet-50. Example images from correctly predicted HV (**a**, **b**), PN– (**c**, **d**) and PN+ (**e**, **f**). First row, original images; second row, Grad-CAM images; third row, Guided Grad-CAM images; and fourth row, occlusion sensitivity images

Figure 3 shows six example CCM images from the test set that were correctly detected, and the resulting Grad-CAM, Guided Grad-CAM and occlusion sensitivity images generated. The attribution maps for correctly detected HV (Fig. 3a,b) highlighted the presence of corneal nerves, focusing on the main nerve segment, emphasised by the Guided Grad-CAM. PN− images that were correctly detected (Fig. 3c,d) had attribution maps which highlighted areas of corneal nerves but displayed shorter segments on the Guided Grad-CAM. Attribution maps from correctly detected PN+ images (Fig. 3e,f) highlighted areas with absence of corneal nerves. Compared with occlusion sensitivity maps, Grad-CAM and Guided Grad-CAM were able to indicate the attribution of the image more clearly. ESM Fig. 1 and ESM Fig. 2 display the attribution maps generated from MobileNet and MobileNetV2, respectively.

## Discussion

CCM is a non-invasive ophthalmic imaging modality which may serve as a bona fide biomarker of diabetic neuropathy [36]. It has been posited as a game changer in the screening and diagnosis of diabetic and peripheral neuropathies [37]. Our study demonstrated two main findings: (1) the successful development of an AI-based algorithm without the need for nerve segmentation; and (2) it established accurate classification of individuals with and without peripheral neuropathy and healthy individuals. This is the first study to utilise an AI-based DLA for the classification of peripheral neuropathy with the addition of attribution methods to provide transparency and explanation of the decision-making process.

A number of studies have developed automated software or neural networks for the segmentation of CCM images [14, 23, 38, 39]. Dabbah et al. [14] proposed an automated system that quantified the nerve fibres and used them as feature vectors to enable classification via random forest and neural network classifiers, producing results that matched the expert manual annotation. Colonna et al. [38] proposed a U-Net-based CNN for automated tracing of corneal nerves, achieving 95% sensitivity compared with the manual tracing. Zhou et al. [23] also developed an improved U-Net architecture, achieving superior results compared with baseline and a super performance with existing DLA for segmentation. Zhao et al. proposed a noise-constrained Retinex model to first enhance the CCM image and used exponential curve estimation as the tortuosity measure to outperform previously used methods, and their results were comparable to human experts [39]. More recently, Mou et al. [40] proposed a curvilinear structure segmentation network validated using six different imaging modalities including CCM, using both 2D and 3D images, outperforming a number of other state-of-the-art algorithms [40].

Previously, Williams et al. [22] presented a novel DLA for estimation of CNFL, which achieved an AUC of 0.83, specificity of 87% and sensitivity of 68% for the diagnosis of peripheral neuropathy. A recent study by Oakley et al. [41] utilised a CNN in macaque CCM images with the advantage of being retrained for additional in vitro [42] and in vivo [43] corneal imaging modalities. For both Williams et al. [22] and Oakley et al. [41], deep learning outperformed ACCMetrics, the current most commonly utilised programme for CNFL estimation. However, the development of such AI-based systems requires the acquisition of large image/datasets with human-graded (ground truth) annotations as a reference standard to train the algorithm [44]. Our study validates the use of an AI-based DLA to diagnose peripheral neuropathy without image segmentation prior to classification. The lack of requirement of manual or automated annotation to train the AI-based DLA allows the utilisation of larger datasets as only unannotated CCM images are required [45]. Without reliance on predetermined features and variables, our method enables the AI-based DLA to learn the features it considers of importance, allowing a more complex image analysis. In our study, two non-neuropathic images which were classified as healthy, suggesting a lack of subclinical small fibre loss, essentially denoting the correct classification was determined (lack of disease) and further adding to the method’s validity.

In general, there is a paucity of studies demonstrating the accurate classification (without segmentation) of peripheral neuropathy based on CCM images. As discussed, our AI-based DLA does not rely on traditional methods of image segmentation. Scarpa et al. [21] also employed an AI-based DLA to classify CCM images without image segmentation utilising a CNN, which analysed three non-overlapping images of each eye per individual, classifying them as either healthy or pathological [21]. Our AI-based DLA achieved comparable results in participants with diabetic neuropathy, but additionally differentiated healthy people from individuals with prediabetes or diabetes without neuropathy, indicating that our AI-based DLA detects early subclinical neuropathy in a real-world clinical setting. Recently, Salahouddin et al. [46] developed a novel automated AI-based analysis system which rapidly quantified CNFL and classified patients with diabetic neuropathy using an adaptive neuro-fuzzy inference system, achieving an AUC of 0.95 (92% sensitivity/80% specificity) for discriminating patients with and without diabetic neuropathy. We propose the instigation of a screening programme for diabetic neuropathy utilising CCM alongside diabetic retinopathy screening [47]. The Food and Drug Administration (FDA) has recently approved the first autonomous AI-based DLA to screen for diabetic retinopathy [48]. In Scotland, an AI-based algorithm was used in a real-world screening service and demonstrated good sensitivity and specificity for detecting high-risk retinopathy, which halved the workload for human graders [49].

Our study was based on a relatively small dataset (*N* = 369 participants), resulting in wide CIs, but nevertheless achieved reasonable classification accuracy. Furthermore, only one image from each participant was used, unlike previous studies [18, 21, 22, 46] which have used multiple images. Indeed, despite defining diabetic neuropathy using the Toronto criteria [27], which rely on abnormal nerve conduction [5], our AI-based DLA, which identifies small fibre pathology known to precede large fibre involvement, still achieved reasonable outcomes. Further refining the model by including additional clinical and demographic data may help to further improve the diagnostic performance. This AI-based DLA needs to be validated in a larger study utilising small fibre measures to identify neuropathy and prospectively in a large-scale clinical population. If validated, cost-effectiveness models need to be established to ascertain its health economics impact.

In conclusion, our AI-based DLA achieved a good classification between HV participants and people with prediabetes and diabetes with and without neuropathy, and the addition of attribution methods aids transparency in the decision making. This AI-based DLA, if validated in a larger study, has considerable potential to be adopted into a screening programme for diabetic neuropathy.

## Supplementary Information

ESM 1 (PDF 229 kb)

## Abbreviations

AI: Artificial intelligence
CCM: Corneal confocal microscopy
CNFL: Corneal nerve fibre length
CNN: Convolutional neural network
DLA: Deep learning algorithm
ENA: Early Neuropathy Assessment
Grad-CAM: Gradient-weighted class activation mapping
HV: Healthy volunteer
PN+: Participants with peripheral neuropathy
PN–: Participants without peripheral neuropathy
