# Supplementary material for: Artificial intelligence utilising corneal confocal microscopy for the diagnosis of peripheral neuropathy in diabetes mellitus and prediabetes
Source: Diabetologia. 2021 Nov 21;65(3):457–66. doi: 10.1007/s00125-021-05617-x (PMC8803718; doi:10.1007/s00125-021-05617-x)
Supplement: Supplementary file 1 — (PDF 229 kb) [file 125_2021_5617_MOESM1_ESM.pdf]

ESM Table 1. Breakdown of patient groups within the training, validation, and test sets

	**Total** (n=369)		
	**Training set** (n=245)	**Validation set** (n=84)	**Test set** (n=40)
**Healthy volunteers**	65	10	15
**Type 1 diabetes**	63	14	11
**Type 2 diabetes**	89	42	10
**Prediabetes**	28	18	4

ESM Table 2. Confusion matrix report from MobileNet in HV, PN− and PN+

True class	Predicted class		
	**HV**	**PN-**	**PN+**
**HV**	15	0	0
**PN-**	5	7	1
**PN+**	2	1	9

HV, healthy volunteers; PN–, no peripheral neuropathy; PN+, peripheral neuropathy.

ESM Table 3. Confusion matrix report from MobileNetV2 in HV, PN− and PN+

True class	Predicted class		
	**HV**	**PN-**	**PN+**
**HV**	13	2	0
**PN-**	4	8	1
**PN+**	1	2	9

HV, healthy volunteers; PN–, no peripheral neuropathy; PN+, peripheral neuropathy.

ESM Table 4. Classification report from MobileNet in HV, PN− and PN+

Class	Recall (Sensitivity)	Precision	F_1-score
HV	1.0 (1.0 – 1.0)	0.68 (0.50 – 0.87)	0.81 (0.67 – 0.93)
PN-	0.54 (0.25 – 0.82)	0.88 (0.57 – 1.0)	0.67 (0.36 – 0.87)
PN+	0.75 (0.46 – 1.0)	0.90 (0.67 – 1.0)	0.82 (0.58 – 0.96)

Note: 95% CIs are given in brackets. HV, healthy volunteers; PN–, no peripheral neuropathy; PN+, peripheral neuropathy.

ESM Table 5. Classification report from MobileNetV2 in HV, PN− and PN+

Class	Recall (Sensitivity)	Precision	F_1-score
HV	0.87 (0.67 – 1.0)	0.72 (0.50 – 0.93)	0.79 (0.60 – 0.92)
PN-	0.62 (0.33 – 0.90)	0.67 (0.36 – 0.92)	0.64 (0.38 – 0.84)
PN+	0.75 (0.46 – 1.0)	0.90 (0.67 – 1.0)	0.82 (0.57 – 0.97)

Note: 95% CIs are given in brackets. HV, healthy volunteers; PN–, no peripheral neuropathy; PN+, peripheral neuropathy.

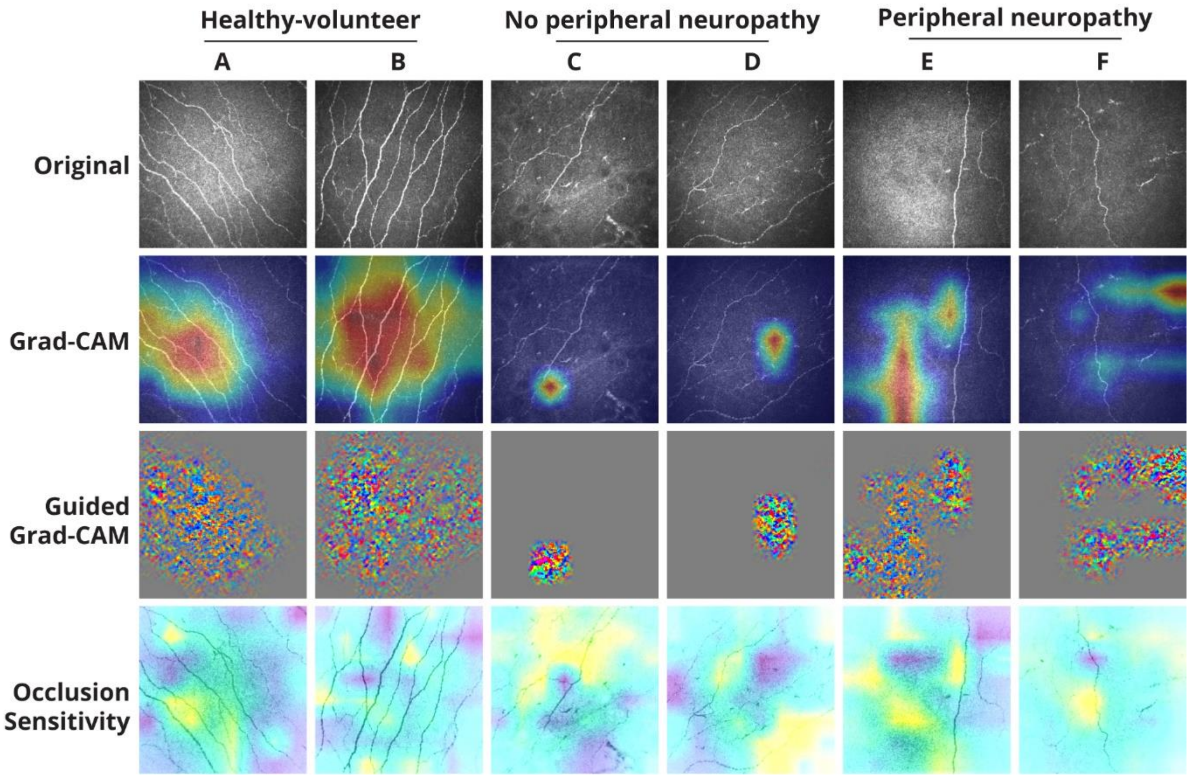

ESM Fig 1. Attribution map results from MobileNet. Example images from correctly predicted healthy volunteer (A,B), no peripheral neuropathy (C,D), and peripheral neuropathy (E,F). First row, original images; second row, Grad-CAM (gradient-weighted class activation mapping) images; third row, Guided Grad-CAM images and fourth row, occlusion sensitivity images.

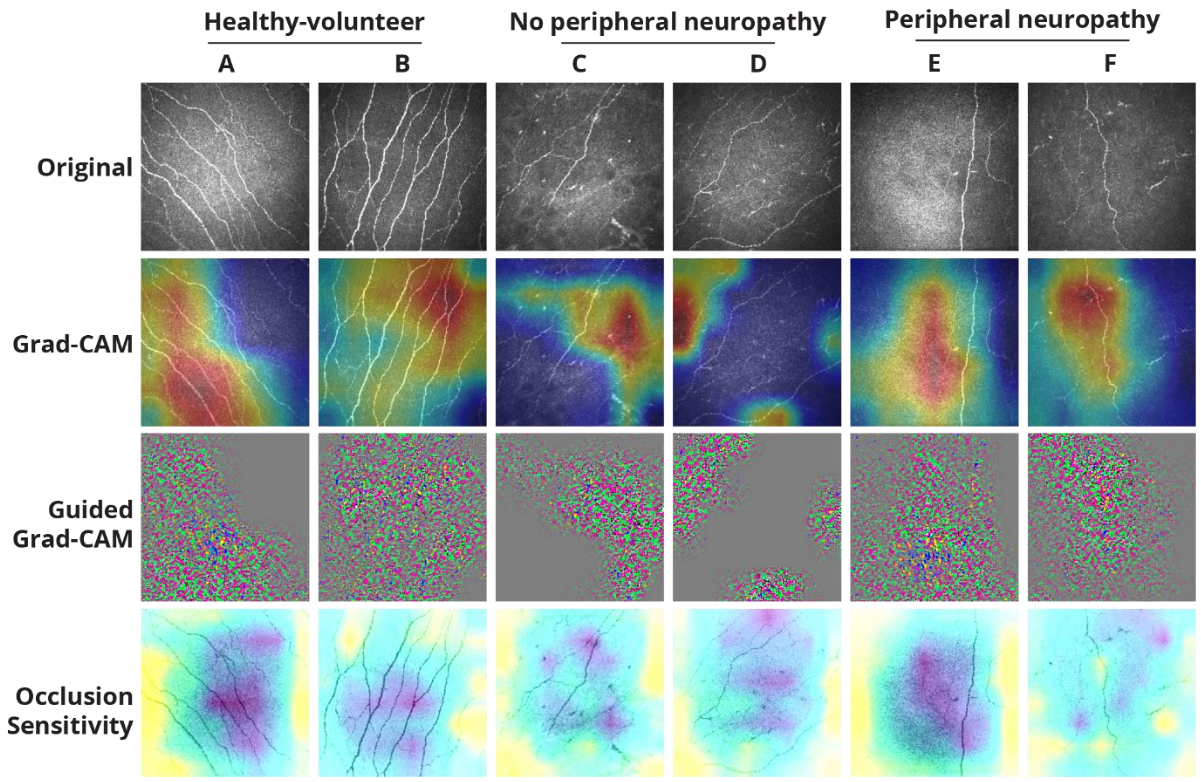

ESM Fig 2. Attribution map results from MobileNetV2. Example images from correctly predicted healthy volunteer (A,B), no peripheral neuropathy (C,D), and peripheral neuropathy (E,F). First row, original images; second row, Grad-CAM (gradient-weighted class activation mapping) images; third row, Guided Grad-CAM images and fourth row, occlusion sensitivity images.
